# Two NAD-linked redox shuttles maintain the peroxisomal redox balance in *Saccharomyces cerevisiae*

**DOI:** 10.1038/s41598-017-11942-2

**Published:** 2017-09-19

**Authors:** Nadal A. Al-Saryi, Murtakab Y Al-Hejjaj, Carlo W. T. van Roermund, Georgia E. Hulmes, Lakhan Ekal, Chantell Payton, Ronald J. A. Wanders, Ewald H. Hettema

**Affiliations:** 1 Department of Molecular Biology, University of Sheffield, Sheffield, UK; 2 Department of Microbiology, College of Veterinary Medicine, University of Basrah, Basrah, Iraq; 3 Laboratory Genetic Metabolic Diseases, Department of Clinical Chemistry, Academic Medical Center, Amsterdam, The Netherlands; 4 School of Life Sciences, University of Lincoln, Lincoln, UK; 5 Present Address: Department of Biology, College of Science, Al Mustansiriyah University, Baghdad, Iraq

## Abstract

In *Saccharomyces cerevisiae*, peroxisomes are the sole site of fatty acid β-oxidation. During this process, NAD^+^ is reduced to NADH. When cells are grown on oleate medium, peroxisomal NADH is reoxidised to NAD^+^ by malate dehydrogenase (Mdh3p) and reduction equivalents are transferred to the cytosol by the malate/oxaloacetate shuttle. The ultimate step in lysine biosynthesis, the NAD^+^-dependent dehydrogenation of saccharopine to lysine, is another NAD^+^-dependent reaction performed inside peroxisomes. We have found that in glucose grown cells, both the malate/oxaloacetate shuttle and a glycerol-3-phosphate dehydrogenase 1(Gpd1p)-dependent shuttle are able to maintain the intraperoxisomal redox balance. Single mutants in *MDH3* or *GPD1* grow on lysine-deficient medium, but an *mdh3/gpd1Δ* double mutant accumulates saccharopine and displays lysine bradytrophy. Lysine biosynthesis is restored when saccharopine dehydrogenase is mislocalised to the cytosol in *mdh3/gpd1Δ* cells. We conclude that the availability of intraperoxisomal NAD^+^ required for saccharopine dehydrogenase activity can be sustained by both shuttles. The extent to which each of these shuttles contributes to the intraperoxisomal redox balance may depend on the growth medium. We propose that the presence of multiple peroxisomal redox shuttles allows eukaryotic cells to maintain the peroxisomal redox status under different metabolic conditions.

## Introduction

Eukaryotic cells are compartmentalised into distinct, membrane-bound organelles which contain their own unique enzyme content and internal milieu. One of these is the peroxisome, a single membrane bound organelle which contains enzymes required for a wide range of reactions and pathways including oxidation reactions resultant in the production of hydrogen peroxide. Some peroxisomal functions such as β-oxidation are widely conserved across almost all eukaryotes whilst other functions may be distinct to certain eukaryotic kingdoms, species or cell types. The importance of correctly localising enzymes to peroxisomes is illustrated by the numerous devastating disorders which arise as a consequence of the mislocalisation of single or multiple peroxisomal enzymes^1^.

**Figure 1:**
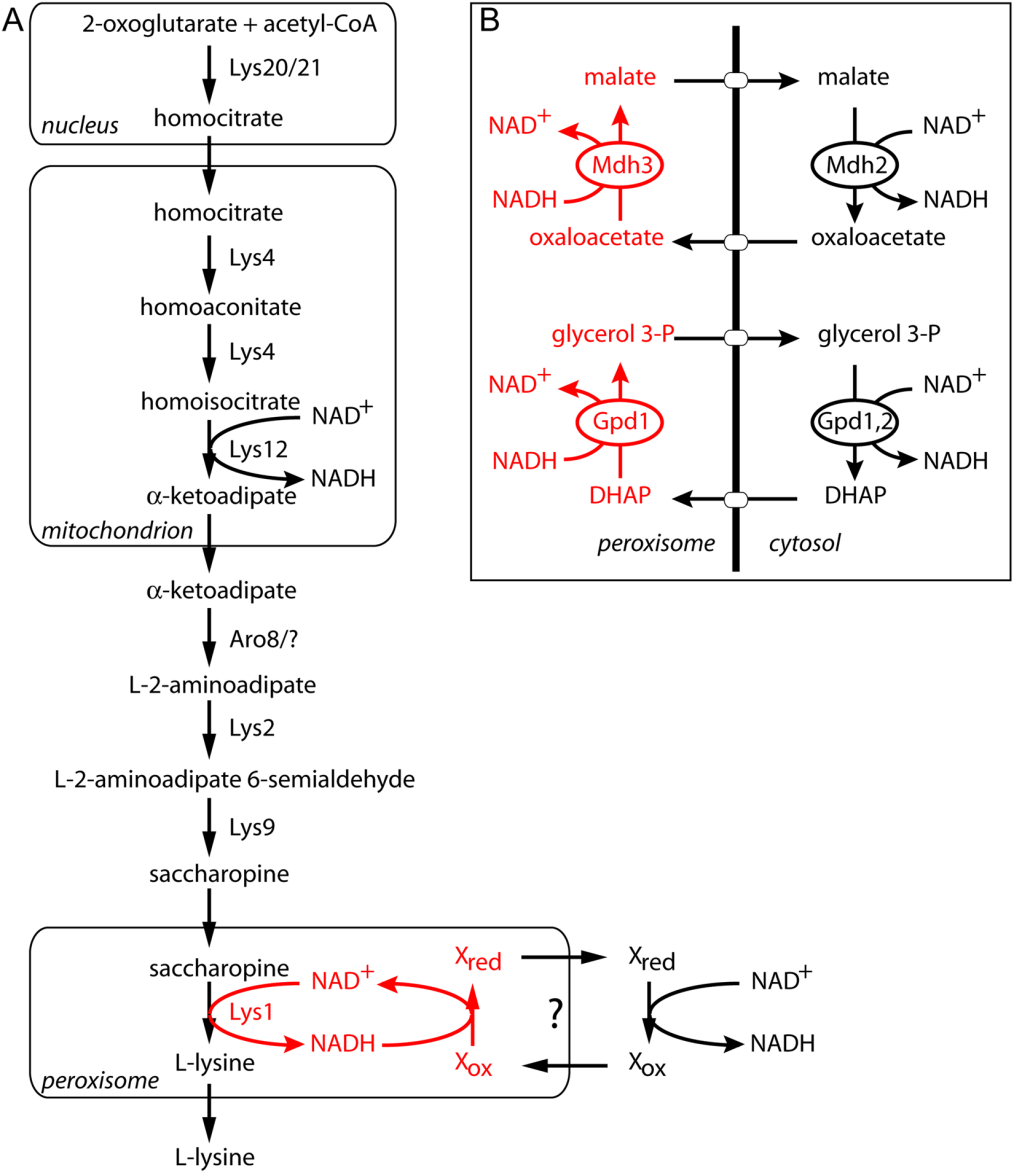
Schematic representation of the intracellular organisation of (**A**) L-lysine biosynthesis in *Saccharomyces cerevisiae*. The subcellular localisations of the enzymes are indicated according to *Saccharomyces cerevisiae* Genome Database (SGD). A generic peroxisomal NADH redox shuttle for reoxidation of NADH to NAD^+^ is depicted in red. (**B**) Diagram of the malate/oxaloacetate shuttle and glycerol-3-phosphate/dihydroxyacetone phosphate shuttle, exchanging peroxisomal NADH for cytosolic NAD^+^. Gpd1p provides the peroxisomal glycerol-3-phosphate dehydrogenase activity. In the cytosol this may be provided by Gpd2p or by cytosolic Gpd1p.

Many metabolic pathways are distributed across multiple subcellular compartments. This organisation is likely to be beneficial to cells as it allows for individual reactions in a pathway to occur within different, favourable micro-environments. Compartmentalisation of reactions allows for additional levels of spatial or temporal control and may offer cellular protection from toxic reaction intermediates or by-products. For example, peroxisomes contribute to the fine-tuning of lysine biosynthesis in *Saccharomyces cerevisiae* and in cells deficient in peroxisomal protein import, mRNA levels of most of the genes involved in lysine biosynthesis are upregulated^2^. In *S. cerevisiae* and many other fungi and euglenoids, L-lysine is produced via the α-aminoadipate pathway (Fig. 1), whereas bacteria, plants and some lower fungi use the diaminopimelate pathway^3^. Based on the localisation of GFP-tagged fusion proteins^4,5^ the pathway for lysine biosynthesis appears to pass through three distinct compartments (nucleus, mitochondrion, cytosol) before it concludes inside the peroxisomal matrix with the enzyme saccharopine dehydrogenase, encoded by *LYS1* (Fig. 1). Metabolic pathways that are distributed across multiple compartments require transport of substrates, co-factors and products between these compartments. For peroxisomes, two types of transport have been described. The first involves transporters that mediate the movement of bulky metabolites and the second type is mediated by passive diffusion of small hydrophilic metabolites of maximally 300–400 Da through channels (porins)^6^.

Although nicotinamide dinucleotides can be transported across the peroxisomal membrane, at least in *Arabidopsis thaliana* ^7,8^, efficient β-oxidation requires redox shuttles to locally reoxidise the NADH produced in the third step of β-oxidation. In *S. cerevisiae*, peroxisomal malate dehydrogenase (Mdh3p) is required to maintain the redox balance inside peroxisomes during growth on fatty acids as the sole carbon source^9,10^. Under these growth conditions, peroxisomes proliferate and expression of β-oxidation enzymes is induced^11^, including Mdh3p^9^. It is unclear whether Mdh3p is required for maintaining the peroxisomal redox balance under other growth conditions. Besides Mdh3p, several additional peroxisomal enzymes may influence the availability of NAD^+^. Glycerol 3-phosphate dehydrogenase 1 (Gpd1p) is a highly abundant enzyme that partially localises to peroxisomes in *S. cerevisiae* even under growth conditions where peroxisome proliferation and β-oxidation are not induced^12^. It has been proposed to regulate the peroxisomal redox status^12,13^ but evidence for this is lacking. Interestingly, glycerol-3-phosphate dehydrogenase activity has also been found to be associated with peroxisomes in mammals and Trypanosomes^14–17^.

We set out to investigate whether Gpd1p contributes to NAD^+^ availability inside peroxisomes in *S. cerevisiae*, under various growth conditions. In agreement with our previous studies, we found that the intraperoxisomal redox balance is maintained predominantly by Mdh3p when cells are grown on oleate medium. We provide evidence that lysine biosynthesis can be maintained by both the Mdh3p- and the Gpd1p-dependent shuttles during growth on glucose medium. We further show that when NAD^+^-linked saccharopine dehydrogenase is mislocalised to the cytosol, lysine biosynthesis occurs independently of the peroxisomal redox shuttles. We conclude that yeast peroxisomes contain two NAD^+^-linked shuttles to maintain the availability of intraperoxisomal NAD^+^ and that the contribution of these shuttles varies between different metabolic conditions.

## Results

**Figure 2:**
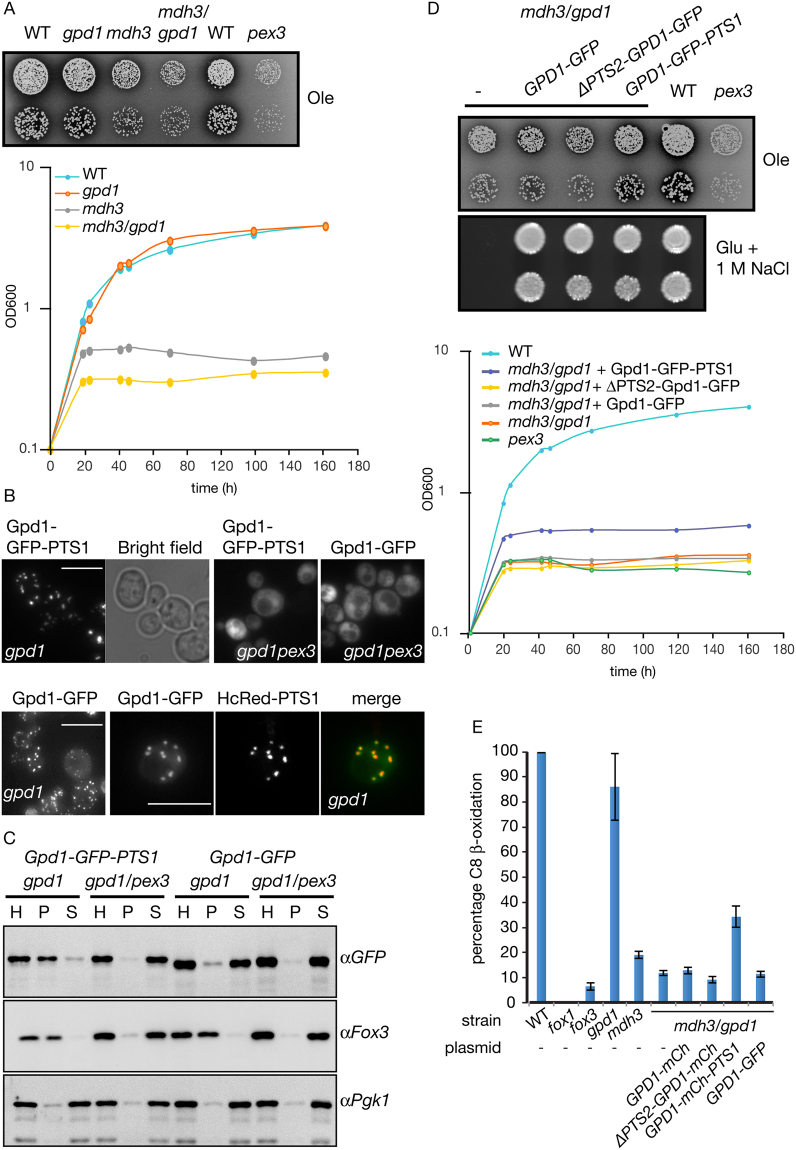
Increased levels of peroxisomal Gpd1p can alleviate the effect of an *MDH3* gene deletion. (**A**) Various *S. cerevisiae* strains were grown on oleate plates or liquid oleate medium for up to 7 days at 30 °C. (**B**) Fluorescence microscopy images of *gpd1Δ* and *gpd1/pex3Δ* cells expressing either Gpd1p-GFP-PTS1 or Gpd1p-GFP and *gpd1Δ* cells co-expressing Gpd1p-GFP and HcRed-PTS1. Bar, 5 μm. Signal for Gpd1p-GFP was enhanced compared to that of Gpd1p-GFP-PTS1. Merge is overlay of GFP and HcRed signals. (**C**) Western blots of subcellular fraction experiments of strains described in (**B**). H, Homogenate, P, 20,000 *g* pellet, S, 20,000 *g* supernatant. Cell equivalents were loaded. (**D**) Strains transformed with the plasmids indicated were grown at 30 °C on oleate plates or glucose/1 M NaCl plates for 7 days or 2 days, respectively. (−), control plasmid. Transformed strains were also grown on liquid oleate medium for 161 h. (**E**) ^14^C-Octanoate β-oxidation activity in intact yeast cells. All strains contained either a control plasmid (−) or expression plasmids encoding different versions of Gpd1p under control of the *GPD1* promoter. Strains were grown overnight on oleate medium. WT cells were normalised to 100%. Three independent experiments were performed. Error bars represent standard error of the mean. WT activity: 4741 +/− 873 pmol/hr/OD_600_.

Gpd1p is partially localised to the peroxisomal matrix but its intraperoxisomal role has remained obscure. It has been suggested that peroxisomal Gpd1p is part of a redox shuttle required to regenerate NAD^+^ formed during fatty acid oxidation. However, peroxisomal malate dehydrogenase (Mdh3p) performs this role^9^. *mdh3Δ* cells are strongly, although not completely, deficient in fatty acid oxidation and show an oleate non-utilising phenotype: limited growth on media containing oleic acid as the sole carbon source (Fig. 2A). *gpd1Δ* cells grow on oleate medium and utilise oleate as a carbon source, this is evident from the clearance of the medium around the growth area (Fig. 2A). Growth of the *mdh3/gpd1Δ* double mutant appears to be slightly more affected than the *mdh3Δ* single mutant. As small growth differences are difficult to judge on oleate containing plates, growth curves were performed which confirmed the growth phenotypes observed on solid media (Fig. 2A). We conclude that Gpd1p is not playing a major role in intraperoxisomal NAD^+^ regeneration during growth on oleate which is in agreement with previous observations^12^.

To test whether Gpd1p could be part of a redox shuttle and function in the regeneration of NAD^+^ inside peroxisomes, we decided to increase the level of Gpd1p in peroxisomes. Gpd1p-GFP is imported into peroxisomes via the PTS2 pathway but this import is rather inefficient^12,13^. By appending Gpd1p-GFP with a peroxisomal targeting signal type 1 (PTS1) import occurs more efficiently^12^. Gpd1p-deficient cells were transformed with plasmids encoding Gpd1p-GFP and Gpd1p-GFP-PTS1 under control of the *GPD1* promoter. These cells were subsequently grown on oleate medium and imaged using identical camera settings. Addition of a PTS1 to Gpd1p-GFP results in a decrease in the level of cytosolic labelling concomitant with an increase in intensity of punctate fluorescence upon addition of the PTS1 (Fig. 2B). There is peroxisomal import of only a small fraction of the total Gpd1p-GFP but a punctate pattern is still visible. This is due to the relatively high concentration of Gpd1p-GFP inside peroxisomes compared to in the cytosol. Confirmation that the punctate fluorescence of Gpd1p-GFP and Gpd1p-GFP-PTS1 is due to peroxisomal localisation is evidenced by their mislocalisation to the cytosol in peroxisome-deficient *pex3Δ* cells. Furthermore, Gpd1p-GFP puncta co-localise with the peroxisomal marker HcRed-PTS1. In addition, homogenates of oleate-grown cells were fractionated by differential centrifugation (Fig. 2C). Gpd1p-GFP-PTS1 fractionates mainly into the 20,000 *g* pellet together with the peroxisomal marker 3-ketoacyl-CoA thiolase (Fox3p) in *gpd1Δ* cells. In contrast, Gpd1p-GFP is mainly recovered in the 20,000 *g* supernatant that includes cytosol and particles with low sedimentation coefficients. In peroxisome-deficient cells (*gpd1/pex3Δ*) both Gpd1p-GFP and Gpd1p-GFP-PTS1 end up in the 20,000 *g* supernatant. These experiments confirm that addition of a PTS1 to Gpd1p-GFP increases its localisation to peroxisomes in oleate grown cells.

*gpd1Δ* cells are sensitive to hyperosmotic growth conditions as they are impaired in the formation and accumulation of glycerol which acts as an osmolyte. As expected, *mdh3*/*gpd1Δ* cells also fail to grow on glucose medium containing 1 M NaCl (Fig. 2D). Expression of GFP tagged versions of Gpd1p rescues this growth defect (Fig. 2D) implying that the fusion proteins are enzymatically active.

We measured β-oxidation activity to further analyse the intraperoxisomal role of Gpd1p. *gpd1Δ* cells are able to oxidise octanoate to near WT levels. Furthermore *mdh3Δ* cells and *mdh3/gpd1Δ* cells displayed a strong β-oxidation defect with the latter mutant being slightly more affected (Fig. 2E). Expression of Gpd1p-GFP in *mdh3/gpd1Δ* cells fails to rescue β-oxidation or growth on oleate to the level of *mdh3Δ* cells. This suggests that insufficient peroxisomal Gpd1p activity is provided by the Gpd1p-GFP expression construct. However, an increase of peroxisomal Gpd1p activity by expression of Gpd1p-GFP-PTS1 partially rescues the growth defect of *mdh3/gpd1Δ* cells on oleate medium (Fig. 2D), and rescues fatty acid β-oxidation intermediate to that of WT and *mdh3Δ* cells (Fig. 2E). We conclude that, although peroxisomal Gpd1p is able to reoxidise NADH to provide intraperoxisomal NAD^+^, the intraperoxisomal redox balance is maintained predominantly by Mdh3p in oleate grown cells.

**Figure 3:**
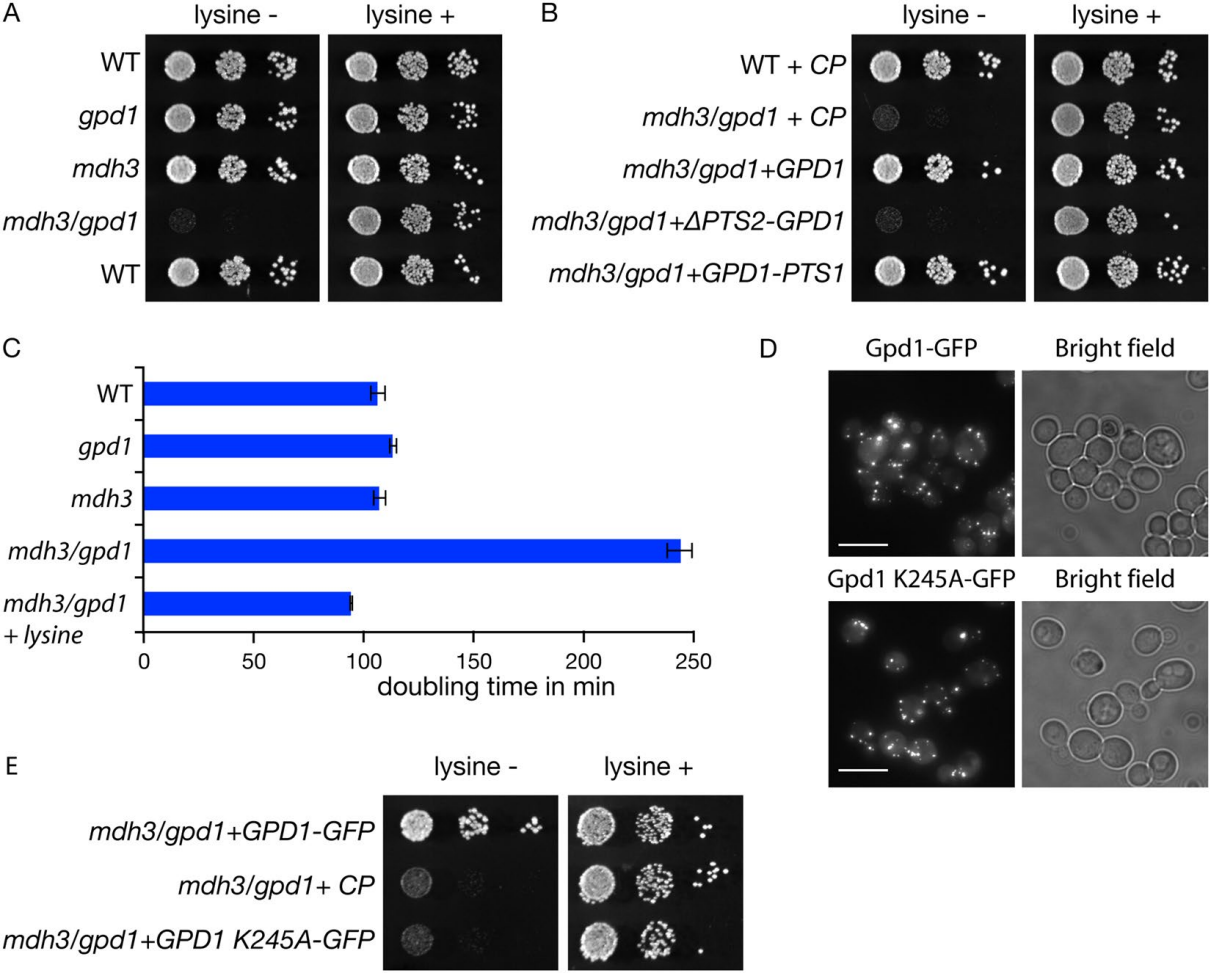
Mdh3p and Gpd1p are involved in lysine biosynthesis. (**A**,**B**,**E**) Growth of the strains indicated on solid complete synthetic glucose medium (CSM) either in the presence or absence of L-lysine for 2 days at 30 °C. Gpd1 versions encoded by plasmids were tagged with GFP. (CP), control plasmid. (**C**) Doubling time of indicated strains during the exponential growth phase in batch cultures on 2% glucose medium lacking lysine. n = 3, error bars indicate standard error of the mean. (**D**) Fluorescence microscopy images of *gpd1Δ* cells expressing either Gpd1p-GFP or Gpd1p K245A-GFP grown on glucose medium. Bar, 5 μm.

*S. cerevisiae* peroxisomes have been functionally linked to lysine biosynthesis^2^ and recently, the enzyme required for the ultimate step in the lysine biosynthesis pathway, saccharopine dehydrogenase, was shown to localise to peroxisomes^5^. Based on a variety of global localisation studies and more detailed analyses, our current model of the spatial organisation of the lysine biosynthetic pathway is summarised in Fig. 1. Saccharopine dehydrogenase, encoded by the *LYS1* gene, requires NAD^+^ for the production of lysine. We tested whether lysine biosynthesis requires a peroxisomal pool of Gpd1p or Mdh3p. Both *mdh3Δ* cells and *gpd1Δ* cells grow well on minimal glucose medium in either the presence or the absence of lysine. Interestingly, an *mdh3/gpd1Δ* double mutant is a lysine bradytroph (Fig. 3A). Both the addition of lysine to this medium or the reintroduction of Gpd1p expression restored growth (Fig. 3B,C). Gpd1p lacking its peroxisomal targeting signal did not restore growth of *mdh3/gpd1Δ* cells in lysine-deficient glucose medium (Fig. 3B). Furthermore, we have previously shown that replacing the active site residue lysine 245 with alanine, has a deleterious effect on activity. Although the mutant is stably expressed^18^ and targeted to peroxisomes to a similar extent as wild type Gpd1p-GFP (Fig. 3D), expression of Gpd1p K245A-GFP was unable to restore lysine biosynthesis in *mdh3/gpd1Δ* cells (Fig. 3E). We conclude that both Mdh3p and peroxisomal Gpd1p activity are required for efficient lysine biosynthesis in glucose grown cells.

**Figure 4:**
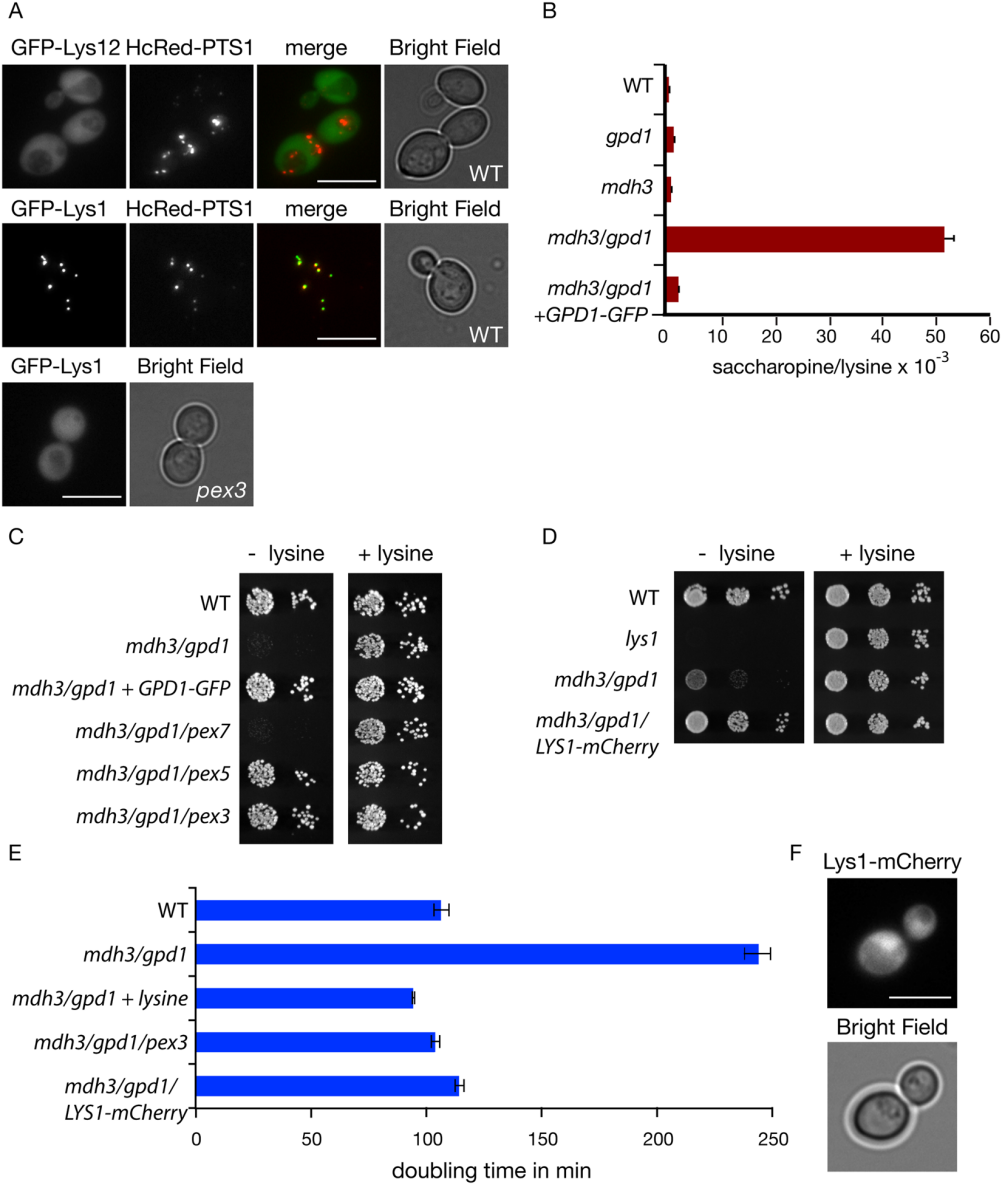
Mdh3p and Gpd1p are required for lysine biosynthesis only when saccharopine dehydrogenase is localised to peroxisomes. (**A**) Fluorescence microscopy of cells expressing GFP-tagged Lys12p or Lys1p with the peroxisomal matrix marker HcRed-PTS1. Bar, 5 μm. (**B**) The ratio of saccharopine/lysine in indicated strains grown overnight on 2% glucose medium lacking lysine. (**C**,**D**) Growth of the strains indicated on solid complete synthetic glucose medium (CSM) either in presence or absence of L-lysine for 2 days at 30 °C. (**E**) Doubling time of indicated yeast strains during the exponential growth phase in batch cultures on 2% glucose medium lacking lysine. n = 3, error bars indicate standard error of the mean. (**F**) Fluorescence microscopy of *mdh3/gpd1Δ* cells with Lys1p tagged in the genome at its C-terminus with mCherry.

Besides saccharopine dehydrogenase, an additional NAD^+^-dependent dehydrogenase (homoisocitrate dehydrogenase encoded by the *LYS12* gene) in the pathway contains the C-terminal tripeptide SRL that resembles the prototypic PTS1^19^ and is identical to the PTS1 of Lys1p. This putative PTS1 is conserved in related yeast species, but Lys12p also contains a typical N-terminal mitochondrial targeting signal (MTS) and Lys12p-GFP localises to mitochondria^4^. Since a PTS1 only acts at the extreme C-terminus of a protein, the use of a C-terminal GFP tag would interfere with peroxisomal targeting. Indeed, tagging proteins at the N-terminus instead of the C-terminus has uncovered many new peroxisomal proteins^5^. We tagged Lys12p at the N-terminus in a yeast expression plasmid and transformed it into WT cells. GFP-Lys12p did not colocalise with the peroxisomal marker HcRed-PTS1, in contrast to GFP-Lys1p in glucose grown cells. The N-terminal tag does interfere with the mitochondrial targeting signal of Lys12p, hence GFP-Lys12p mislocalised to the cytosol (Fig. 4A). Based on these observations we postulate that the lysine biosynthesis defect in *mdh3/gpd1Δ* cells is a consequence of a decrease in saccharopine dehydrogenase activity.

Changes in the redox state in a particular cellular compartment can be determined by measuring changes in the ratio of the concentrations of the oxidised and reduced metabolites of a compartment-specific NAD^+^-linked dehydrogenase^20^. A disruption of the peroxisomal NAD^+^/NADH ratio as a consequence of a block in the redox shuttles should lead to an increase in the Lys1p substrate/product ratio. Indeed, the saccharopine/lysine ratio increases from 0.633 ± 0.03 × 10^−3^ in WT cells to 51.4 ± 1.98 × 10^−3^ in *mdh3/gpd1Δ* cells, an increase of more than 80 fold. In *mdh3Δ* and *gpd1Δ* cells the ratios were only slightly increased compared to WT (1.07 ± 0.05 × 10^−3^ and 1.51 ± 0.02 × 10^−3^, respectively) (Fig. 4B).

Localisation of Lys1p to peroxisomes is not essential for lysine biosynthesis as peroxisome-deficient cells are not lysine bradytrophs^2^. We hypothesised that if we mislocalised Lys1p to the cytosol in *mdh3/gpd1Δ* cells, the cytosolic pool of NAD^+^ would support Lys1p activity and lysine biosynthesis would be restored. In agreement with our hypothesis, growth on lysine-deficient medium was restored in cells that lack peroxisomes (*mdh3/gpd1/pex3Δ* cells) and in cells that are deficient in import of PTS1-containing proteins (*mdh3/gpd1/pex5Δ* cells). In *mdh3/gpd1/pex7Δ* cells, where PTS2 import is selectively blocked, no growth restoration occurs (Fig. 4C) as Lys1p is imported via the PTS1 pathway. Subsequently, Lys1p was tagged with mCherry at the C-terminus in the genome, which inactivates its PTS1. This results in its cytosolic localisation (Fig. 4F) and as we hypothesised, restores growth on lysine-deficient medium (Fig. 4D,E). This indicates that a decrease of saccharopine dehydrogenase activity (Lys1p-activity) causes lysine bradytrophy in *mdh3/gpd1Δ* cells. We conclude that two independent shuttles operate to maintain the availability of peroxisomal NAD^+^. Each shuttle can be sufficiently active in the absence of the other shuttle to support lysine biosynthesis to a level that does not increase cell doubling time in glucose medium.

## Discussion

We set out to discern the role of glycerol-3-phosphate dehydrogenase inside peroxisomes. Our experimental data implicates this enzyme in the reoxidation of intraperoxisomal NADH to NAD^+^. Peroxisomal NAD^+^ is required to support NAD^+^-dependent dehydrogenation reactions. Gpd1p is not the only enzyme inside peroxisomes that can fulfil this role. Malate dehydrogenase encoded by the *MDH3* gene was previously shown to perform this role when cells are growing on fatty acids as the sole carbon source. Under these growth conditions, cells depend on fatty acid β-oxidation for growth. In *mdh3Δ* cells, β-oxidation is blocked at the level of 3-hydroxyacyl-CoA dehydrogenase^9^. By increasing the peroxisomal pool of Gpd1p, we were able to partially overcome this block which illustrates that Gpd1p can substitute for Mdh3p, but normally does not.

Our further studies showed that Gpd1p together with Mdh3p are required for lysine biosynthesis, in particular to maintain saccharopine dehydrogenase activity inside peroxisomes. Two NAD^+^-dependent dehydrogenases in the lysine biosynthesis pathway (Lys1p and Lys12p) contain a C-terminal tripeptide that fits the PTS1 consensus. Indeed GFP-Lys1p localises to peroxisomes but GFP-Lys12p does not. The presence of a putative PTS1 in Lys12p is intriguing and could point to it being imported into peroxisomes under conditions different from the ones used in this study. Alternatively, the context of the C-terminus of Lys12p could be preventing its recognition by the PTS1 receptor. Two additional observations support our conclusion that during growth on glucose medium, saccharopine dehydrogenase (Lys1p) is the only lysine biosynthetic enzyme that is dependent on the availability of intraperoxisomal NAD^+^. Firstly, by mislocalising Lys1p to the cytosol in *mdh3/gpd1Δ* cells, lysine biosynthesis is restored and secondly, in *mdh3/gpd1Δ* cells the substrate/product ratio for Lys1p is 80 fold increased.

Why *S. cerevisiae* contains two distinct parallel pathways to reoxidise intraperoxisomal NADH is still unclear. The presence of multiple peroxisomal redox shuttles appears to be widespread throughout the eukaryotic kingdom. For instance, in addition to glycerol 3-phosphate dehydrogenase, mammalian liver peroxisomes contain malate dehydrogenase and lactate dehydrogenase that have been suggested to act as redox shuttles. However, evidence for a role *in vivo* is lacking^21–24^. *T. brucei*, in its mammalian host, relies exclusively on glycolysis for energy production. Under this condition, the glycerol 3-phosphate dehydrogenase/DHAP shuttle regenerates NAD^+^^15^. In the insect or procyclic form of *T. brucei*, intra-glycosomal NAD^+^ is regenerated via three different pathways, of which the glycerol 3-phosphate dehydrogenase/DHAP shuttle appears to be a back-up pathway^25,26^.

The requirement for multiple redox shuttles is also seen when considering the distinct shuttles which are operational between the mitochondrial matrix and the cytosol. These shuttles allow exchange of reduction equivalents under varying growth conditions^27^. Likewise, the presence of multiple peroxisomal shuttles may allow for maintenance of the NAD^+^/NADH ratio under a wide range of metabolic conditions with the contribution of each shuttle differing as conditions vary. For instance, the malate/oxaloacetate shuttle is important during growth of *S. cerevisiae* on oleate, a condition where Mdh3p and the glyoxylate cycle enzymes (including Mdh2p) are induced and oxaloacetate will be available. Under these gluconeogenic conditions, either the DHAP availability or the level of Gpd1p inside peroxisomes may be too low for an effective operation of the glycerol 3-phosphate/DHAP shuttle. In contrast, both shuttles operate when cells are grown in glucose medium. The dehydrogenase of one of the shuttles uses the PTS1 import route whilst the dehydrogenase of the other shuttle is imported via the PTS2 pathway. This may further allow for flexibility in the regulation of the two shuttles.

Expression of Gpd1p-GFP in glucose grown *mdh3/gpd1Δ* cells restored growth on lysine deficient medium, normalised the saccharopine/lysine ratio and supported growth under hyperosmotic conditions. This suggests that the Gpd1p-GFP expression construct is fully functional under these growth conditions. However, on oleate medium it did not seem to be fully active. The reason for this is unclear but may reflect a decrease in peroxisomal import or limited activity of the fusion protein that only becomes apparent under certain growth conditions.

Our study emphasises a central role of peroxisomes in the regulation of NAD^+^ metabolism. Gpd1p co-imports the nicotinamidase Pnc1p^13,18,28^. Its role inside peroxisomes is unclear as was the role of Gpd1p. Further experiments are under way to investigate whether the co-import of Pnc1p with Gpd1p is linked to the regulation of the peroxisomal NAD^+^ pool.

## Experimental procedures

### Yeast strains, media and growth conditions

Cells were grown at 30 °C in any of the following media: YPD (1% yeast extract, 2% peptone, 2% glucose), minimal media (YM2) for the selection of the uracil (URA3) prototrophic marker (2% glucose, 0.17% yeast nitrogen base without amino acids, 0.5% ammonium sulphate, 1% casamino acids) or minimal media (YM1) for the selection of all prototrophic markers except uracil (2% glucose, 0.17% yeast nitrogen base without amino acids, 0.5% ammonium sulphate). For analysis of growth characteristics, cells were grown on 2% glucose in complete synthetic medium lacking lysine (Formedium, UK). In some cases, as indicated in figure legends, L-lysine was added at 6 mg/ml. Liquid oleate media constituted of 0.5% potassium phosphate buffer pH 6.0, 0.1% yeast extract, leu^−^ drop out (Formedium), 0.17% yeast nitrogen base without amino acids, 0.1% oleate, 0.2% Tween 40. For growth curves, cell density (OD_600_) was measured after washing samples twice in water.

*S. cerevisiae* strains used in this study are listed in supplementary Table 1. BY4741 or BY4742 wild type strains and deletion mutants marked with KanMX cassette sequence were obtained from Euroscarf. Any strains created in this study were modified from the BY4741 or BY4742 using the HphMX4 cassette^29^ and selected on YPD containing 300 µg/ml Hygromycin B (Melford, UK).

### Plasmids

Most of the plasmids used in this study, were constructed by gap repair^30^ in Ycplac derivatives^31^. *LYS1* and *LYS12* were amplified by PCR containing 18 nucleotide flanking regions identical to each side of the intended insertion site and inserted into a Tpi1 promoter driven N-terminal expression vector derived from Ycplac33. All *GPD1* expression constructs contained 523 bp upstream from the ATG as their promoter regions and have been described previously^18^. In brief, Gpd1p-mCherry-PTS1 was constructed by fusion of mCherry-SKL to the C-terminus of Gpd1p. ∆PTS2-Gpd1p was constructed by deletion of the first twenty-one amino acids from the N-terminus and reintroduction of a start codon. Peroxisomal marker plasmid HcRed-PTS1 was described previously^32^.

### Image acquisition

Image acquisition and processing was performed as described previously^33^. Cells were analysed with a microscope (Axiovert 200 M; Carl Zeiss MicroImaging, Inc.) equipped with an Exfo X-cite 120 excitation light source, band pass filters (Carl Zeiss MicroImaging, Inc. and Chroma Technology Corp.), an α-Plan-Fluar 100x/1.45 NA, Plan-Apochromat 63x/1.4 NA or A-plan (Carl Zeiss MicroImaging, Inc.) and a digital camera (Orca ER; Hamamatsu). Image acquisition was performed using Volocity software (Improvision). Fluorescence images were collected as 0.5 μm Z-stacks using exposures of up to 200 ms, merged into one plane in Openlab and processed further in Photoshop (Adobe). Bright field images were collected in one plane.

### Subcellular fractionation

Cells grown overnight on oleate medium were converted to spheroplasts with Zymolyase 20T (5 mg/g cells). The spheroplasts were washed twice in ice cold 1.2 M sorbitol/5 mM 2(*N*-morpholino)ethane sulfonic acid (MES) (pH 6)/1 mM EDTA/1 mM KCl before resuspension in 0.65 M sorbitol/5 mM MES (pH 6)/1 mM EDTA/1 mM KCl (fractionation buffer) containing 1 mM PMSF and Protease inhibitor cocktail. Cell breakage was achieved by careful resuspension of spheroplasts and incubation on ice for 10 min. Intact cells and nuclei were removed by centrifugation (800 *g* for 10 min). From this homogenate (Hom), 1 ml was transferred to a new Eppendorf tube and centrifuged at 20,000 *g* for 20 min. The supernatant (S) was collected and the pellet (P) was resuspended in 1 ml fractionation buffer. Equivalent volumes of these fractions were analysed by SDS–PAGE and immunoblotting. Monoclonal anti-GFP antibody was obtained from Roche (11814460001), and anti-Pgk1 22C5 from Invitrogen. Anti-yeast Thiolase was a kind gift from Ben Distel. Secondary antibody was HRP-linked Goat anti-mouse polyclonal (BioRad 1706516). Blots were blocked in 2% (w/v) fat-free Marvel™ milk in TBS-Tween 20 (50 mM Tris-HCl (pH 7.5), 150 mM NaCl, 0.1% (v/v) Tween 20). Immunoreactive proteins were detected by enhanced chemiluminescence (Biological Industries) and blots were imaged with a G box (Syngene).

### β-Oxidation activity measurements

β-oxidation assays in intact yeast cells were performed as described previously^9^ and optimized for the pH and the amount of protein. Oleate-grown cells were washed in water and resuspended in 0.9% NaCl (OD_600_ = 1.0). Aliquots of 20 μl of cell suspension were used for β-oxidation measurements in 200 μl of 50 mM MES (pH 6.2) and 0.9% (w/v) NaCl supplemented with 10 μM [1-^14^C]-octanoate. After 1 hr at 28 °C, the incubation was stopped with 50 μl 2.6 M perchloric acid (PCA). Subsequently, [1-^14^C]-CO_2_ was trapped with 500 μl 2 M NaOH. The ^14^C labelled β-oxidation products were then collected after extracting the acidified material with cloroform/methanol/heptane and quantified in a liquid scintillation counter. The quantification of the rate of fatty acid oxidation was measured by the summation of the amount of [1-^14^C]-CO_2_ and ^14^C-labelled β-oxidation products. Results are presented as percentage relative activity to the rate of oxidation of wild type cells.

### Saccharopine/lysine ratio measurement

Yeast cells were grown overnight on 2% glucose and intact cells were harvested at 4 °C. 0.5 OD_600_ units of cells were resuspended in 50 μl PBS and transferred to a 1.5 ml Eppendorf tube. 0.5 ml of 100% ACN plus 20 µl of internal standard mixture (containing 32 nmol d4-lysine) were added and homogenized for 15 sec by vortexing. Samples were centrifuged for 10 min at 4 °C at a speed of 12,000 *g*. The supernatant was transferred to a 4 ml glass vial and evaporated under a stream of nitrogen at 40 °C. After evaporation, 110 µl of 0.01% heptafluorobutyric acid was added to dissolve the residue and mixed by vortexing. This suspension was transferred to a Gilson vial for HPLC-MS/MS analysis as described previously^34^. For the calculation of AA concentrations, 50 µl standards containing 0, 30, 60, 125 or 250 μM lysine or saccharopine in PBS were added to the internal standard (20 µl, same composition as mentioned above) and analyzed as described above.

## Electronic supplementary material

Supplementary information
