# Supplementary material for: Two NAD-linked redox shuttles maintain the peroxisomal redox balance in Saccharomyces cerevisiae
Source: Sci Rep. 2017 Sep 19;7:11868. doi: 10.1038/s41598-017-11942-2 (PMC5605654; doi:10.1038/s41598-017-11942-2)

Supplementary information

Two NAD-linked redox shuttles maintain the peroxisomal redox balance in *Saccharomyces cerevisiae*

Nadal A. Al-Saryi[1,2], Murtakab Y Al-Hejjaj[1], Carlo W. T. van Roermund[3], Georgia E. Hulmes[1], Lakhan Ekal[1], Chantell Payton[4], Ronald J. A. Wanders[3] and Ewald H. Hettema[1]

1 Department of Molecular Biology, University of Sheffield, UK

2 Current address, Department of Biology, College of Science, Al Mustansiriyah University, Bagdad, Iraq

3 Laboratory Genetic Metabolic Diseases, Department of Clinical Chemistry, Academic Medical Center, Amsterdam, The Netherlands

4 School of Life Sciences, University of Lincoln, UK

Corresponding author:
E.H. Hettema
Email: e.hettema@sheffield.ac.uk

Supplementary table 1. Yeast strains used in this study

Strain	Source
BY4741 MATa *his3-1 leu2-0 met15-0 ura3-0*	Euroscarf
BY4742 MATα *his3-1 leu2-0 lys2-0 ura3-0*	Euroscarf
BY4741 *fox1::kan^r*	Euroscarf
BY4741 *fox3::kan^r*	Euroscarf
BY4741 *gpd1::kan^r*	Euroscarf
BY4741 *mdh3::SchizHIS5*	This study
BY4741 *pex3::kan^r*	Euroscarf
BY4741 *gpd1::kan^r, mdh3::SchizHIS5*	This study
BY4741 *pex3::hph^r, gpd1::kan^r, mdh3::SchizHIS5*	This study
BY4741 *pex5::hph^r, gpd1::kan^r, mdh3::SchizHIS5*	This study
BY4741 *pex7::hph^r, gpd1::kan^r, mdh3::SchizHIS5*	This study
BY4741 *gpd1::kan^r, mdh3::SchizHIS5 LYS1-mCherry::hph^r*	This study

Supplementary figure 1

Full sized western blots from which the compilation in Fig. 2 was made

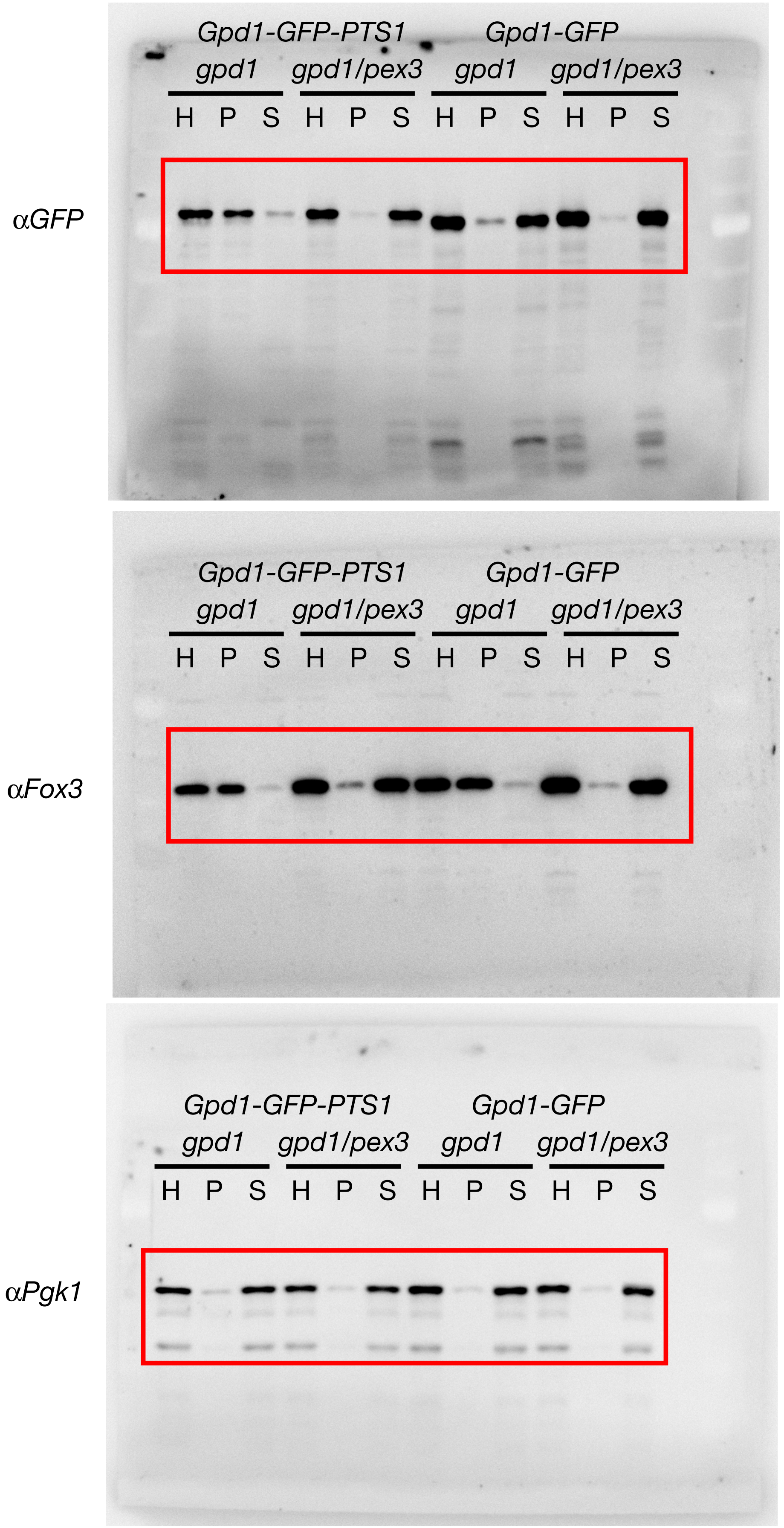

Supplement: Supplementary file 1 — Supplementary information [file 41598_2017_11942_MOESM1_ESM.pdf]
